# Association of Low Level Viremia with Inflammation and Mortality in HIV-Infected Adults

**DOI:** 10.1371/journal.pone.0026320

**Published:** 2011-11-02

**Authors:** Abigail Eastburn, Rebecca Scherzer, Andrew R. Zolopa, Constance Benson, Russell Tracy, Tri Do, Peter Bacchetti, Michael Shlipak, Carl Grunfeld, Phyllis C. Tien

**Affiliations:** 1 University of California San Francisco, San Francisco, California, United States of America; 2 Department of Veterans Affairs Medical Center, San Francisco, California, United States of America; 3 Stanford University, Stanford, California, United States of America; 4 University of California San Diego, San Diego, California, United States of America; 5 University of Vermont, Burlington, Vermont, United States of America; 6 Roche Molecular Diagnostics, Pleasanton, California, United States of America; Tulane University, United States of America

## Abstract

**Background:**

Whether HIV viremia, particularly at low levels is associated with inflammation, increased coagulation, and all-cause mortality is unclear.

**Methods:**

The associations of HIV RNA level with C-reactive protein (CRP), fibrinogen, interleukin (IL)-6 and mortality were evaluated in 1116 HIV-infected participants from the Study of Fat Redistribution and Metabolic Change in HIV infection. HIV RNA level was categorized as undetectable (i.e., “target not detected”), 1–19, 20–399, 400–9999, and ≥10,000 copies/ml. Covariates included demographics, lifestyle, adipose tissue, and *HIV*-related factors.

**Results:**

HIV RNA level had little association with CRP. Categories of HIV RNA below 10,000 copies/ml had similar levels of IL-6 compared with an undetectable HIV RNA level, while HIV RNA ≥10,000 copies/ml was associated with 89% higher IL-6 (p<0.001). This association was attenuated by ∼50% after adjustment for CD4+ cell count. Higher HIV RNA was associated with higher fibrinogen. Compared to an undetectable HIV RNA level, fibrinogen was 0.6%, 1.9%, 4.5%, 4.6%, and 9.4% higher across HIV RNA categories, respectively, and statistically significant at the highest level (p = 0.0002 for HIV RNA ≥10,000 copies/ml). Higher HIV RNA was associated with mortality during follow-up in unadjusted analysis, but showed little association after adjustment for CD4+ cell count and inflammation.

**Conclusion:**

HIV RNA ≥10,000 copies/ml was associated with higher IL-6 and fibrinogen, but lower levels of viremia appeared similar, and there was little association with CRP. The relationship of HIV RNA with IL-6 was strongly affected by CD4 cell depletion. After adjustment for CD4+ cell count and inflammation, viremia did not appear to be substantially associated with mortality risk over 5 years.

## Introduction

Chronic inflammation and increased hemostasis in HIV-infected persons are thought to be a consequence of viral replication or persistence and its associated immune activation. Increased levels of inflammatory and coagulation markers have been associated with increased mortality in HIV-infected participants [1], [2]. In the Study of Fat Redistribution and Metabolic Change in HIV infection (FRAM), we previously reported that markers of inflammation and coagulation remain important predictors of death even at higher CD4+ cell counts [2].

Whether low levels of viremia predispose to increased inflammation is unclear. Many patients on antiretroviral therapy (ART) with undetectable viremia by conventional assays have been shown to have persistent low level viremia when a more sensitive research assay (limit of detection <3.5copies/ml) was used [3]. Studies [1], [4], [5] that have used conventional HIV RNA assays suggest that inflammation may persist despite having undetectable HIV RNA. In one small study, elite controllers (HIV-infected persons with undetectable HIV RNA in the absence of ART) had higher levels of the inflammatory marker C-reactive protein (CRP) than HIV-uninfected controls [4]. In the SMART trial [5], levels of CRP, interleukin (IL)-6 and D-dimer remained elevated in persons with HIV infection even after suppression of HIV RNA with ART. The SMART trial also showed that IL-6 and D-dimer were strongly associated with mortality even in HIV-infected adults who achieved viral suppression [1]. These studies used HIV RNA assays with a lower limit of detection of 75 copies/ml [4] and 400 copies/ml [1].

Using a recently approved ultrasensitive HIV RNA assay, we evaluated the association of viremia with CRP, the pro-inflammatory cytokine IL-6, and the clotting protein fibrinogen in HIV-infected participants of the FRAM study. We then examined whether HIV RNA levels were associated with increased all-cause mortality risk after adjusting for demographic and cardiovascular risk factors, CD4+ cell count, and markers of inflammation and coagulation. We hypothesized that even very low levels of viremia would be associated with ongoing inflammation.

## Methods

### Ethics Statement

The Committee on Human Research at the University of California, San Francisco approved the Office of the Principal Investigator at the University of California, San Francisco to oversee all data collection activities for this multicenter study at 16 sites across the United States, for both FRAM exams. The approved protocol includes the investigation of markers of infection and inflammation and their association with adipose tissue changes, metabolic perturbations and their long term consequences. Approval for data collection activities by the Institutional Review Board (IRB) at each respective site were also obtained including the University Hospitals Case Medical Center IRB, the Tufts Medical Center/Tufts University IRB, and the Stanford IRB and the Research and Human Subjects Review Committee of Santa Clara Valley Medical Center (the full name of all data collection sites and their IRB are listed in the Appendix S1). Written informed consent was obtained from all participants. All clinical investigation was conducted according to the principles expressed in the Declaration of Helsinki.

### Study Population

FRAM is a large, nationally representative, multicenter study of HIV infection, originally designed to evaluate the prevalence and correlates of changes in fat distribution and metabolic parameters. The methods, design, and sample characteristics of the FRAM cohort have been described previously [6]. Briefly, between June 2000 and September 2002, 1183 HIV-infected men and women from 16 geographically diverse sites across the United States were enrolled in FRAM, with a follow-up exam conducted approximately five years later (FRAM2) (2004–2007). Retention outcomes for participants enrolled in the first exam have been reported [7]. At the second exam, 922 HIV-infected participants were known to be alive or dead; vital status could not be determined for the remaining 261 HIV-infected participants. Linkage to the National Death Index was not possible because of institutional review board and patient confidentiality issues. The second exam included 581 HIV-infected participants recruited from those seen at the first exam.

### Study measurements

#### HIV viral load

As previously reported [6] , at both visits, blood specimens were analyzed in a single centralized laboratory (Covance, Indianapolis, Indiana), including a standard lipid panel, CD4+ lymphocyte count and HIV RNA (Roche COBAS® AMPLICOR HIV-1 Monitor Test; lower limit of quantitation: 400 copies/ml). For this study, a single 1-ml aliquot sample of frozen plasma for each visit (FRAM1 and FRAM2) was retested using the FDA-approved COBAS® AmpliPrep/COBAS® TaqMan® HIV-1 Test, version 2.0 (Roche Molecular Diagnostics, RMD). The approved lower limit of quantitation and detection for the assay is 20 copies/ml. For values below 20 copies/ml, clinical results are reported as “<20 copies/ml detectable” or “<20 copies/ml undetectable.” For the purposes of our study, RMD provided actual values below the limit of 20 copies/ml, that ranged from 0 to 19. A value of 0 indicates that the test did not detect copies of virus (i.e. “target not detected”), but does not necessarily mean that the true HIV RNA level was 0 copies/ml given the imprecision of the test at extremely low levels of virus. The two timepoints (FRAM1 and FRAM2) were used separately to predict inflammation.

#### Inflammatory Biomarkers

As described previously [8], [9], CRP and fibrinogen were quantitatively measured in frozen sera and plasma, respectively (i.e., stored at −70 degrees Celsius and not previously thawed) at both the first and second FRAM examination using the BNII nephelometer from Dade Behring (Deerfield, IL) which utilizes a particle-enhanced immunonephelometric assay. The lower limit of detection for the ultrasensitive CRP assay used in FRAM was 0.16 mg/L. The inter-assay coefficient of variation (CV) for CRP ranged from 3.7% to 4.5%. The intra- and inter-assay CV for fibrinogen was 2.7% and 2.6% respectively. IL-6 was measured from frozen sera collected at the second FRAM examination by ultrasensitive enzyme-linked immunosorbent assay (Quantikine HS Human IL-6 Immunoassay; R&D Systems). The lower limit of detection was 0.16 pg/mL. The intra-assay CV for IL-6 was 6.3%. Based on 3–4 controls per assay, the inter-assay CV ranged from 7% to 15% for IL-6. CRP, fibrinogen, and IL-6 were measured at the Laboratory for Clinical Biochemistry Research at the University of Vermont or the University of California, San Francisco.

#### Covariates

Covariates were collected at both examinations. Clinical information was collected using standardized questionnaires, laboratory, and anthropometric measurement protocols. The following information was considered for inclusion in multivariable models of CRP, fibrinogen, and IL-6: demographic characteristics (age, sex, and race), lifestyle factors (physical activity, alcohol use, adequate food intake, and smoking), regional and total adipose tissue (as described previously [10]), and HIV-related factors (HIV RNA level, CD4+ cell count, AIDS [11], duration of HIV infection, and HCV infection [defined by detectable HCV RNA]).

In models of mortality, covariates from the first exam included demographics, traditional cardiovascular disease risk factors (diabetes, smoking, blood pressure, and HDL and total cholesterol), CD4+ cell count, and markers of inflammation (CRP and fibrinogen). Multiple imputation using the Markov chain Monte Carlo method was used to impute missing covariates [12].

### Statistical analysis

Of the 1183 HIV-infected participants enrolled in FRAM, 1116 had available HIV RNA data and were included in the analysis. We compared demographic and clinical characteristics at baseline across levels of HIV RNA using the Kruskal-Wallis test for continuous variables and a chi-squared test for categorical variables. We discretized HIV RNA as follows: 0, 1–19, 20–399, 400–9999, and ≥10,000 copies/mL.

We tested the linearity assumption for the relationship of HIV RNA with inflammatory markers and mortality by examining generalized additive models [13]. We used multivariable linear regression to evaluate the association of HIV RNA at each exam with CRP, fibrinogen, and IL-6. To account for repeated measures from the two exams (FRAM1 and FRAM2), linear mixed models with random intercepts and slopes were used to evaluate the association of HIV RNA with CRP and fibrinogen. IL-6 data was only available in FRAM2. All analyses were adjusted using inverse probability weighting to address the impact of selection bias [14].

We adjusted for covariates in stages, first including demographics variables (age, gender, and race), then adding lifestyle factors, regional adipose tissue depots, and finally adding HIV-related factors to the model. The final multivariate-adjusted models were built separately for each outcome (CRP, fibrinogen, and IL-6) using stepwise regression with a p-value of 0.05 or less for entry and retention of covariates.

As in previous analyses [2], [15], we used multivariable logistic regression to evaluate the association of baseline HIV RNA with cumulative 5-year mortality. Since the exact dates of death were unknown, those who died provided left-censored observations, meaning that death was only known to have occurred sometime before the contact attempt at approximately 5 years of follow-up. We therefore used logistic regression with an offset term for follow-up time, rather than Cox proportional hazards regression as our primary analysis, because this form of regression is appropriate for left-censored events. Follow-up time was defined as elapsed time from baseline to follow-up exam or last contact. To account for those with missing vital status, we also adjusted estimates using an inverse probability weighting approach by modeling the participant's probability of having known death status [14]. The inverse of this probability was then used as a weight applied to persons with known vital status in the logistic regression analysis of death.

All analyses were conducted using the SAS system, version 9.2 (SAS Institute, Inc., Cary, North Carolina).

## Results

At baseline, the overall median age of the study participants was 42 years. Women comprised 30% of the study population. The overall median value for HIV RNA was 280 copies/ml. Table 1 presents the baseline demographic and clinical characteristics of the 1,116 HIV-infected participants stratified by HIV RNA level (0 copies/ml, 1–19 copies/ml, 20–399 copies/ml, 400–10,000 copies/ml, and ≥10,000 copies/ml). Participants with higher HIV RNA were more often African-American, had lower visceral adipose tissue (VAT) and higher leg subcutaneous adipose tissue (SAT), and were more often smokers, injection drug users, and HCV-infected. Those with higher HIV RNA also were less often HAART users and had lower rates of ART adherence and lower CD4+ cell counts. Current smoking was less frequent in those with 0 copies/ml compared to those with 1–19 copies/ml (29% vs. 43%, p = 0.0019). Median CD4+ cell count appeared to be slightly higher in those with 0 copies/ml compared to those with 1–19, but the difference did not reach statistical significance (484 vs. 453, p = 0.27).

**Table 1:** Baseline demographic and clinical characteristics of HIV-infected participants by HIV viral load category.

	Viral Load (copies)					P-value
	0	1–19	20–399	400-10K	>10K	
N	210	137	236	237	296	
Age (y)	43.5 (38.0–50.0)	42.0 (37.0–49.0)	42.5 (37.0–49.0)	43.0 (36.0–48.0)	42.0 (36.5–47.0)	0.18
Female	65 (31.0%)	36 (26.3%)	58 (24.6%)	74 (31.2%)	97 (32.8%)	0.24
Race						
Caucasian	117 (55.7%)	79 (57.7%)	130 (55.1%)	85 (35.9%)	124 (41.9%)	**<.0001**
Black	63 (30.0%)	42 (30.7%)	81 (34.3%)	117 (49.4%)	140 (47.3%)	
Hispanic	23 (11.0%)	13 (9.5%)	21 (8.9%)	28 (11.8%)	26 (8.8%)	
Other	7 (3.3%)	3 (2.2%)	4 (1.7%)	7 (3.0%)	6 (2.0%)	
BMI (kg/m^2^)	25.2 (22.4–28.7)	24.7 (22.7–26.9)	24.2 (22.3–26.7)	25.1 (22.5–29.1)	24.5 (21.7–27.9)	**0.046**
VAT (kg)	2.1 (1.0–3.1)	1.9 (0.9–3.0)	1.4 (0.6–2.6)	1.5 (0.6–2.4)	1.1 (0.5–2.2)	**<.0001**
Leg SAT (kg)	3.0 (1.9–5.4)	2.6 (1.9–4.8)	3.2 (1.9–4.9)	3.4 (2.1–5.4)	3.6 (2.2–6.1)	**0.0019**
Smoking Status						
Current	61 (29.0%)	59 (43.1%)	93 (39.4%)	105 (44.3%)	136 (45.9%)	**0.0001**
Past	40 (19.0%)	33 (24.1%)	66 (28.0%)	45 (19.0%)	60 (20.3%)	
Never	109 (51.9%)	45 (32.8%)	77 (32.6%)	87 (36.7%)	100 (33.8%)	
Alcohol	87 (41.4%)	49 (35.8%)	92 (39.0%)	101 (42.6%)	123 (41.6%)	0.71
IDU	29 (13.8%)	26 (19.0%)	48 (20.3%)	49 (20.7%)	82 (27.7%)	**0.0047**
HCV	32 (15.2%)	25 (18.2%)	54 (22.9%)	61 (25.7%)	72 (24.3%)	**0.045**
Current HAART	195 (92.9%)	128 (94.1%)	197 (83.8%)	154 (65.0%)	177 (60.2%)	**<.0001**
ART Adherent (y/n)	167 (81.9%)	110 (82.1%)	176 (81.9%)	130 (68.8%)	127 (67.6%)	**<.0001**
Current CD4 (cells/uL)	484 (315–660)	453 (275–617)	395 (262–578)	378 (255–528)	189 (77–346)	**<.0001**

Data above are summarized as median (IQR) or number (percent). Analysis is restricted to those with non-missing inflammatory markers.

BMI = body mass index, VAT = visceral adipose tissue, SAT = subcutaneous adipose tissue, IDU = injection drug user, HCV = hepatitis C virus, HAART = highly active antiretroviral therapy, ART = antiretroviral therapy.

### Association of continuous and categorized HIV RNA levels with CRP

There appeared to be little overall association of HIV RNA level with CRP in unadjusted analysis (p = 0.99, test for linear association). Examination of generalized additive models suggested some evidence of non-linearity (p = 0.054), with higher predicted levels of CRP among those with very high levels of viremia (>100,000 copies/ml). However, the variation in CRP was very wide at these very high levels of viremia, and only 86 study participants had levels of HIV RNA above 100,000 copies/ml.

**Figure 1:**
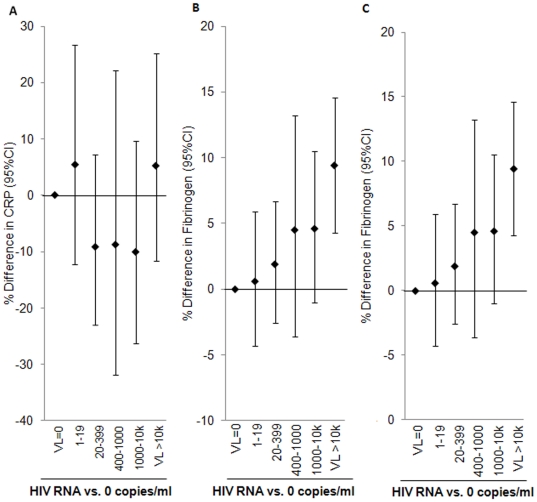
Multivariable-adjusted association of HIV RNA with (A) CRP*, (B) IL-6^†^, (C) fibrinogen^¥^. * (A) Estimates from multivariable-adjusted model controlling for gender, age, race, smoking, physical activity, VAT, Arm SAT, nadir CD4, and HCV status. ^†^ (B) Estimates from multivariable-adjusted model controlling for gender, age, race, smoking, and current CD4. ^¥^ (C) Estimates from multivariable-adjusted model controlling for gender, age, race, smoking, alcohol, upper trunk SAT, nadir CD4, HIV duration, and HCV status.

Therefore, we examined the association of categorized HIV RNA levels with CRP (Table 2 and Figure 1A) using multivariable linear regression analysis. After adjustment for demographics, lifestyle factors, and adipose tissue, any HIV RNA level still showed little association with CRP. Further adjustment for HIV-related factors resulted in little change in the association of HIV RNA with CRP (Figure 1A).

**Table 2:** Association of discretized HIV RNA levels with CRP, IL-6, and fibrinogen in all HIV-infected participants.

		Outcome		
*Model*	Level of Viral Load	CRP	IL-6	Fibrinogen
		%Estimate (95% CI)	%Estimate (95% CI)	%Estimate (95% CI)
Demographics + Lifestyle + Adipose Tissue				
	VL 1–19 vs. VL = 0	4.8 (−13.0, 26.3) p = 0.62	0.8 (−25.7, 36.7) p = 0.96	0.5 (−4.6, 5.8) p = 0.86
	VL 20–399 vs. VL = 0	−9.1 (−23.1, 7.5) p = 0.26	2.0 (−22.6, 34.5) p = 0.89	1.7 (−2.9, 6.5) p = 0.48
	VL 400-1K vs. VL = 0	−11.9 (−34.4, 18.2) p = 0.40	3.4 (−42.4, 85.9) p = 0.91	3.2 (−4.8, 12.0) p = 0.44
	VL 1K-10K vs. VL = 0	−12.0 (−27.9, 7.5) p = 0.21	−9.4 (−38.9, 34.2) p = 0.62	2.4 (−3.0, 8.2) p = 0.39
	VL>10K vs. VL = 0	9.5 (−8.2, 30.5) p = 0.31	**89.2 (40.2, 155.4) p<.0001**	**10.0 (4.9, 15.4) p<.0001**
Demographics + Lifestyle + Adipose Tissue + HIV-related				
	VL 1–19 vs. VL = 0	5.4 (−12.3, 26.7) p = 0.57	0.1 (−26.0, 35.5) p = 0.99	0.6 (−4.3, 5.9) p = 0.81
	VL 20–399 vs. VL = 0	−9.2 (−23.0, 7.2) p = 0.25	0.7 (−23.5, 32.6) p = 0.96	1.9 (−2.6, 6.7) p = 0.41
	VL 400-1K vs. VL = 0	−8.8 (−31.8, 22.1) p = 0.54	−2.5 (−45.6, 74.8) p = 0.93	4.5 (−3.6, 13.2) p = 0.29
	VL 1K-10K vs. VL = 0	−10.1 (−26.3, 9.7) p = 0.29	−14.7 (−42.5, 26.4) p = 0.43	4.6 (−1.0, 10.5) p = 0.11
	VL>10K vs. VL = 0	5.2 (−11.6, 25.2) p = 0.57	**48.9 (5.8, 109.6) p = 0.022**	**9.4 (4.3, 14.6) p = 0.0002**

CRP and Fibrinogen are analyzed using linear mixed models, with adjustment for time (baseline and year 5 data).

IL-6 is analyzed using year 5data only.

### Association of continuous and categorized HIV RNA levels with IL-6

Increasing HIV RNA level was associated with IL-6 in unadjusted analysis (p = 0.0031, test for linear association). Examination of generalized additive models suggested evidence of non-linearity (p = 0.045), with a steeper slope seen among those with higher levels of viremia.

In the multivariable analysis of categorized HIV RNA levels, after adjustment for demographic, lifestyle factors, and adipose tissue, participants with HIV RNA levels below 10,000 copies/ml had levels of IL-6 that were similar to those with zero copies (Figure 1B and Table 2). By contrast, those with HIV RNA above 10,000 copies/ml had 89% higher IL-6 (p<.0001 versus those with zero copies). This association was attenuated by approximately 50% after further adjustment for CD4+ cell count (p = 0.022 versus those with zero copies).

### Association of continuous and categorized HIV RNA levels with fibrinogen

Increasing HIV RNA level was more strongly associated with fibrinogen in unadjusted analysis (p<.0001, test for linear association). Examination of generalized additive models suggested evidence of non-linearity (p = 0.0048), with a steeper slope seen among those with higher levels of viremia (especially ≥10,000 copies/mL).

In multivariable analysis of categorized HIV RNA levels, after adjustment for demographic, lifestyle factors, and adipose tissue, increasing HIV RNA was associated with higher fibrinogen (Table 2 and Figure 1C). Results were similar after further adjustment for HIV-related factors.

### Interaction and sensitivity analyses

Because we found that the association of HIV RNA with IL-6 was attenuated by CD4+ cell count, we tested for CD4 by HIV RNA interaction for each inflammatory marker. There were no statistically significant interactions on CRP, IL-6, or fibrinogen. As expected, we found that IL-6 and fibrinogen were highest in those with HIV RNA ≥10,000 copies/ml and CD4+ cell count <200 (data not shown).

In order to examine whether these associations persisted when limited to those on ART, we performed a sensitivity analysis excluding those not on ART. All associations were similar (data not shown).

### Association of HIV RNA level with all-cause mortality

**Figure 2:**
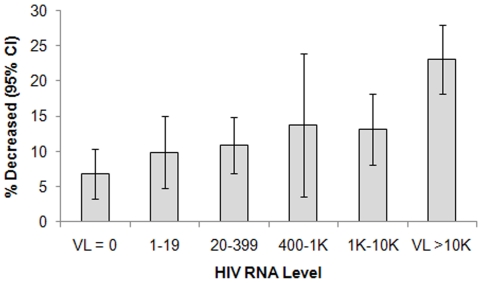
Prevalence of five-year all-cause mortality by HIV RNA level at baseline. Association of baseline HIVRNA (per 10-fold increase) with mortality: Demographic-adjusted: OR = 1.39 (95%CI: 1.26 to 1.55), p<.0001 Fully-adjusted: OR = 0.99 (95%CI: 0.87 to 1.13), p = 0.90 Fully adjusted model includes demographics, traditional CVD risk factors (smoking, diabetes, blood pressure, total and HDL cholesterol), CD4+ cell count, CRP, and fibrinogen.

In unadjusted analysis, five-year all-cause mortality progressively increased with increasing HIV RNA level (p<.0001, Figure 2). The prevalence of death ranged from 6.8% among those with zero HIV RNA copies to 23% among those with ≥10,000 copies/ml. In demographic-adjusted analysis, the odds of mortality increased by 39% for every 10-fold increase in HIV RNA (p<.0001).

However, the association of HIV RNA with mortality was attenuated after adjustment for CD4+ cell count (OR: 1.07, 95% CI: 0.95, 1.21, p = 0.23), and there appeared to be no association after further adjustment for cardiovascular risk factors and inflammation (OR: 0.99, p = 0.90).

Controlling for duration of ART use did not change the association of HIV RNA with mortality (OR = 1.01, 95%CI: 0.89–1.15, p = 0.91). There were no statistically significant interactions of HIV RNA with CRP, fibrinogen, or CD4+ cell count on mortality.

## Discussion

Our study is one of the first to examine the association of low level viremia with inflammatory biomarkers and mortality in a large, nationally representative cohort of HIV-infected adults which allowed for a fully developed multivariable analysis. Contrary to our hypothesis, we found little association of low level viremia with levels of CRP, IL-6 and fibrinogen. Rather, there was little association of any HIV RNA level with CRP, and only HIV RNA level ≥10,000 copies/ml had a relatively strong association with IL-6 and fibrinogen levels.

We made several other noteworthy observations. First, we found that increasing HIV RNA was progressively associated with higher fibrinogen levels, whereas those with HIV RNA level <10,000 copies/ml did not appear to have progressively higher IL-6 levels. Second, the association of increasing viremia with higher fibrinogen did not appear to be attenuated by immune status, whereas the association with IL-6 was strongly attenuated by CD4+ cell count. These findings suggest that the mechanisms by which HIV affects inflammatory and coagulation markers are different. Finally, after adjustment for CD4+ cell count, and inflammatory and coagulation markers, HIV RNA did not appear to be associated with mortality. A recent study from the ATHENA cohort found that low level (HIV RNA 50–400 copies/ml) and high level viremia (HIV RNA >400 copies/ml) were associated with 1.6-fold and 1.5-fold higher risks of death (respectively) compared with undetectable viremia, but the associations did not reach statistical significance, and the study did not adjust for inflammatory and coagulation markers [16].

Our finding that low level viremia had little association with CRP, fibrinogen, and IL-6 was somewhat unexpected. Data from small studies of specific populations of HIV-infected patients suggest a possible association based on other indices of inflammation [17], [18], [19]. In one study of 23 patients on combination ART for a median of 84 months [18], residual HIV replication <50 copies/ml was observed, and in those with poor immunologic response to ART (i.e. gain of <200 CD4 cells), the residual viremia was associated with activation of CD4+ and CD8+ T-cells. In two other small studies [17], [19], HIV controllers (i.e., those with undetectable virus using conventional assays despite the absence of ART) demonstrated abnormal immune responses. In one, cellular HIV RNA and DNA levels were associated with greater %CD38+ HLA-DR+ CD4+ and HIV *gag* responsive T cells^17^. In the other, low level viremia was associated with higher HIV-1- neutralizing antibody levels^19^. By contrast, a recent study of 127 HIV-infected persons found that low level viremia (<50 copies/ml) had little association with markers of immune activation, and had no statistically significant association with CRP, IL-6 or D-dimer [20].

We observed a significant association of viremia with fibrinogen and IL-6 at levels well above the lower limit of detection for conventional HIV RNA assays. Our findings should be compared to data from the SMART trial [1]. In that study, changes in IL-6 and D-dimer levels were measured in 132 previously suppressed subjects, stratified by HIV RNA category one month after discontinuing ART (<400, 401–10,000, 10,000–50,000, and ≥50,000 copies/ml). That study found significant increases in IL-6 and D-dimer with increasing HIV RNA level. However, only the increase in patients whose HIV RNA rose above 50,000 copies/ml one month after discontinuation appeared appreciably greater than those who remained suppressed (<400 copies/ml).

Our study found that the association of HIV RNA with IL-6 was strongly attenuated after adjustment for CD4+ cell count suggesting that immunosuppression is an important determinant of IL-6 levels. Those with both high viremia and low CD4+ cell count were most likely to have high IL-6 levels. In the SMART trial, IL-6 levels were a strong predictor of mortality. By contrast, the association of HIV RNA with fibrinogen showed little attenuation after adjusting for CD4+ cell count, suggesting that immunosuppression does not significantly affect fibrinogen levels. We previously found that the association of fibrinogen with increased mortality risk was also independent of CD4+ cell count [2]. It is therefore of interest that we observed that greater HIV RNA levels did not predict substantially increased mortality in models that included both CD4+ cell count and fibrinogen.

Finally, the reasons for the lack of a significant association of HIV RNA with CRP are unclear. We previously reported that the median CRP in our cohort [9] was well below 3 mg/L. In the general population, CRP levels above 3 mg/L are considered high risk for cardiovascular disease [21]. HIV-infected participants in our cohort had higher median CRP and fibrinogen levels than controls [8], [9]. Similar to a recent study that compared HIV-infected participants from the SMART trial with control participants from the Coronary Artery Risk Development in Young Adults (CARDIA) Study [5], we found that median IL-6 levels were higher in HIV-infected than control participants (1.11 vs. 0.88 pg/ml).

There are limitations to our study. First, because of the nature of our cohort study, we were not able to assess low level viremia from a very large volume of plasma or using a single copy assay, and therefore the reported values at ranges <20 copies/ml may not be accurate. However, this new assay was strongly correlated with the COBAS® AMPLICOR HIV-1 Monitor Test (lower limit of detection: 400 copies/ml) originally used in our cohort. Furthermore, the distribution of clinical characteristics by HIV RNA category (even at very low levels of virus) was in the expected direction. Second, our study was limited by the frequency of sampling, as viremia was assessed at only two timepoints, and by the limited number of inflammation and coagulation markers studied (although the select markers studied allowed comparison with similar markers examined in prior studies). Third, IL-6 was not measured at the first FRAM exam, and so analyses of IL-6 are cross-sectional and limited to participants who enrolled in FRAM 2. Fourth, death and loss- to-follow up after the first FRAM exam may have contributed to bias in the participants enrolled in the second FRAM exam. However, we used inverse probability weighting to mitigate any potential bias from those who did not enroll in the second exam. Fifth, we did not have information for the cause of death. Finally, as with all observational studies, our findings are subject to possible unmeasured confounding.

We conclude that there is little association of low level viremia with levels of CRP, fibrinogen, and IL-6. Our finding that there was little association of any level of HIV RNA with CRP raises the possibility that CRP may not be a reliable marker of inflammation that is caused by ongoing HIV replication or persistence. However, HIV RNA levels ≥10,000 copies/ml were strongly associated with fibrinogen and IL-6. The relationship of HIV RNA with IL-6 (but not fibrinogen) was strongly affected by CD4 depletion. After adjustment for CD4+ cell count and inflammation, there was no apparent association of HIV RNA level with mortality. Our findings suggest that the mechanisms by which HIV infection induces inflammation, influences coagulation, and causes mortality are complex. Additional study of mechanistic pathways including markers of microbial translocation is needed.

## Supporting Information

Appendix S1 Data collection sites.
(DOCX)
