# Supplementary material for: Association of Low Level Viremia with Inflammation and Mortality in HIV-Infected Adults
Source: PLoS One. 2011 Nov 2;6(11):e26320. doi: 10.1371/journal.pone.0026320 (PMC3206804; doi:10.1371/journal.pone.0026320)
Supplement: Appendix S1 — Data collection sites. (DOCX) [file pone.0026320.s001.docx]

**Appendix S1.**

Data in this manuscript were collected by the Fat Redistribution and Metabolic Changes study (FRAM) with data collection sites (Principal Investigators) at University Hospitals of Cleveland (Barbara Gripshover, MD) approved by the University Hospitals Case Medical Center Investigational Review Board (IRB); Tufts University (Abby Shevitz, MD (deceased) and Christine Wanke, MD) approved by the Tufts Medical Center/Tufts University IRB; Stanford University (Andrew Zolopa, MD) approved by the Stanford IRB and the Research and Human Subjects Review Committee of Santa Clara Valley Medical Center; University of Alabama at Birmingham (Michael Saag, MD) approved by the University of Alabama at Birmingham’s IRB for Human Use; Johns Hopkins University (Joseph Cofrancesco Jr., MD and Adrian Dobs, MD) approved by the Johns Hopkins Medicine IRBs; University of Colorado Health Sciences Center (Lisa Kosmiski, MD and Constance Benson, MD) approved by the Colorado Multiple Institutional Review Board; University of North Carolina at Chapel Hill (David Wohl, MD and Charles van der Horst, MD*) approved by the University of North Carolina at Chapel Hill Biomedical IRB; University of California at San Diego (Daniel Lee, MD and W. Christopher Mathews, MD*) approved by the UCSD School of Medicine IRB; Washington University (E. Turner Overton, MD and William Powderly, MD) approved by the Washington University Human Studies Committee; VA Medical Center, Atlanta (David Rimland, MD) approved by the Emory IRB; University of California at Los Angeles (Judith Currier, MD) approved by the UCLA Medical IRB1; VA Medical Center, New York (Michael Simberkoff, MD) approved by the Manhattan/Brooklyn VAMC IRB; VA Medical Center, Washington DC (Cynthia Gibert, MD) approved by the DC VAMC IRB; St Luke’s-Roosevelt Hospital Center (Donald Kotler, MD and Ellen Engelson, PhD) approved by the St Luke’s Roosevelt Hospital IRB; Kaiser Permanente, Oakland (Stephen Sidney, MD) approved by the Kaiser Permanente Northern California Health Services IRB; University of Alabama at Birmingham (Cora E. Lewis, MD) approved by the University of Alabama at Birmingham’s Institutional Review Board for Human Use.

*involved in FRAM1 only
